# The effects of action-based predictions in early visual cortex

**DOI:** 10.1016/j.isci.2026.117074

**Published:** 2026-08-18

**Authors:** Bianca M. van Kemenade, Lars F. Muckli

**Affiliations:** 1 Center for Psychiatry, Justus Liebig University Giessen, Klinikstrasse 36, 35392 Giessen, Germany; 2 Centre for Cognitive Neuroimaging (CCNi), School of Psychology and Neuroscience, University of Glasgow, 62 Hillhead Street, Glasgow, UK

**Keywords:** voluntary action, visual perception, early visual cortex, fMRI

## Abstract

Voluntary action typically results in reduced sensitivity to sensory action outcomes. The forward model theory proposes that this is due to neural suppression. However, this theory has recently been challenged by the pre-activation account, sharpening, and opposing process theory. In this fMRI study, we compared these theories by using univariate and multivariate analyses. Participants performed a visual orientation discrimination task on two sequential gratings, which were presented automatically (passive condition) or triggered by button press (active condition). Decoding of predicted stimulus orientation from early visual cortex activity prior to stimulus presentation was significantly above chance in both active and passive conditions. During stimulus presentation, actively generated stimuli elicited larger blood-oxygen-level-dependent (BOLD) responses. However, both decoding accuracy and the timing of the BOLD responses did not differ between conditions. These results differ from the forward model’s predicted sensory attenuation, the pre-activation account’s earlier BOLD response, and the enhanced precision of the sharpening hypothesis. Instead, they mostly fit the opposing process theory, which posits pre-activation in both conditions. However, the stronger BOLD response for actively generated stimuli is not predicted by any existing theory, implicating additional mechanisms, such as heightened attention or motor-related enhancement.

## Highlights

• Action-based and general sensory predictions can be decoded from early visual cortex
• Active and passive conditions can be cross-decoded, suggesting similar mechanisms
• Voluntary action leads to increased BOLD response in early visual cortex
• Results are mostly in line with opposing process theory

## Graphical abstract

**Figure:**
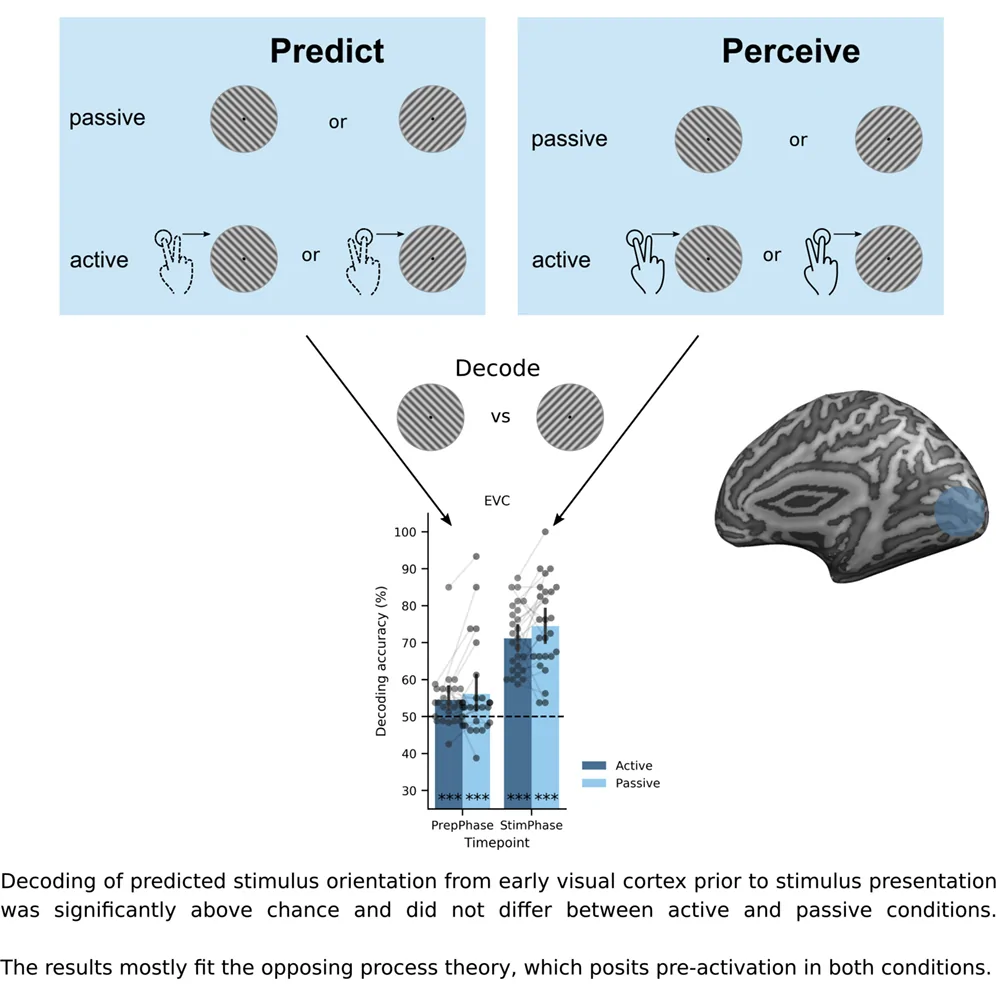

## Teaser

Behavioral neuroscience; Sensory neuroscience

## Introduction

Our actions produce sensory outcomes, contributing substantially to the constant stream of sensory input that we encounter every day. It was proposed decades ago that we suppress sensory consequences of our own action using predictions generated by an internal forward model.^1^ According to this theory, this internal forward model uses the efference copy, a copy of the motor command, to generate predictions about the sensory outcomes of our action.^2^^,^^3^ These predictions are then used to cancel out sensory action consequences, or generate prediction errors in the case of a mismatch between prediction and sensory feedback. This mechanism is thought to aid us in guiding our actions, learn new motor skills, save resources, and distinguish self from other.^4^^,^^5^ In line with this idea are numerous studies that show reduced neural activity elicited by self-generated stimuli compared to identical but externally generated stimuli.^6^^,^^7^^,^^8^^,^^9^^,^^10^ For many years, this theory has shaped our thinking about sensorimotor mechanisms. However, in recent years, this theory has been challenged by three alternative theories. First of all, the pre-activation account posits that suppression of self-generated stimuli is not due to a true dampening of neural activity, but rather a pre-activation of sensory areas.^11^ The idea is that due to this pre-activation, the baseline activity prior to stimulus onset is increased, making it harder to distinguish the actual stimulus activity from the baseline activity, leading to an apparent suppression during stimulus presentation. However, evidence for this account using neuroimaging techniques is lacking. Secondly, studies on sensory predictions have suggested that the apparent suppression induced by sensory expectations, so-called “expectation suppression”,^12^ may instead be due to a sharpening of the underlying neuronal populations. This neural sharpening may resemble suppression when using univariate analysis but can be detected using multivariate pattern analysis showing better decoding of expected stimuli.^13^ It has been proposed that action also leads to neural sharpening,^14^ but, to our knowledge, no one has directly compared active with passive conditions using this approach to confirm that the effects of action on neural activity can be specifically attributed to neural sharpening. Lastly, the recently proposed “opposing process theory” suggests that action-based predictions and general predictions about our environment are based on the same mechanism and involve different time scales.^15^ According to this theory, any prediction about an upcoming event first pre-activates neurons tuned to the expected features. During stimulus presentation, “cancellation” may occur in the form of boosting the unexpected event compared with the expected event but only when the unexpected event is likely to be informative. This theory unites the fields of action-based and general sensory predictions, but as it was proposed only very recently, the support for this theory is still preliminary. Evidence for pre-activation of neurons tuned to expected features exists in the perception literature,^16^^,^^17^ but evidence for neural pre-activation for action-based predictions, as well as a supposed similarity between these two types of predictions, is lacking. In addition to these theories, the possibility that there are no effects of predictions on perception should be considered in a null theory.

In this fMRI study, we combined univariate and multivariate analysis techniques both prior to and during stimulus presentation in order to directly test and compare all theories. This strategy allowed us to test for changes in both overall neural amplitude and representational content during both prediction and perception phases (see Table 1). If action cancels out sensory outcomes through dampening, as the forward model theory suggests, we would expect both reduced neural activity and reduced decoding accuracy for self-generated stimuli. If action instead sharpens neural responses, we would expect reduced neural activity but increased decoding accuracy for self-generated stimuli. If action modulates neural activity through pre-activation, we would expect increased activity and increased decoding accuracy during preparation and suppressed neural activity and decreased decoding accuracy during stimulus presentation. Lastly, if action-based and general sensory predictions follow the same principles as outlined in the opposing process theory, we would expect a pre-activation of expected units through significant above-chance decoding of the upcoming stimulus during preparation, with no differences between self-generated and externally generated conditions. During stimulus presentation, we would expect no differences in activity or decoding accuracy, at least when using highly predictable stimuli. The null theory would predict no differences between active and passive conditions for any of the time points and no pre-activation of expected units during preparation.

**Table 1:** The models’ hypotheses about activity level and decodability of visual features during the preparation phase and in response to stimulus presentation

	Preparatory activity	Preparatory decoding	Stimulus activity	Stimulus decoding
Forward model	−	−	A+ < P+	A+ < P+
Sharpening	−	−	A+ < P+	**A+ > P+**
Pre-activation	**A+ > P**	**A+ > P**	A+ < P+	A+ < P+
Opposing process	A+ = P+	**A+ = P+**	A+ = P+	A+ = P+
Null	A = P	**A = P**	A+ = P+	A+ = P+

The + symbols indicate significant above-baseline activity/above-chance decoding. Marked in bold are unique predictions.

## Results

**Figure 1:**
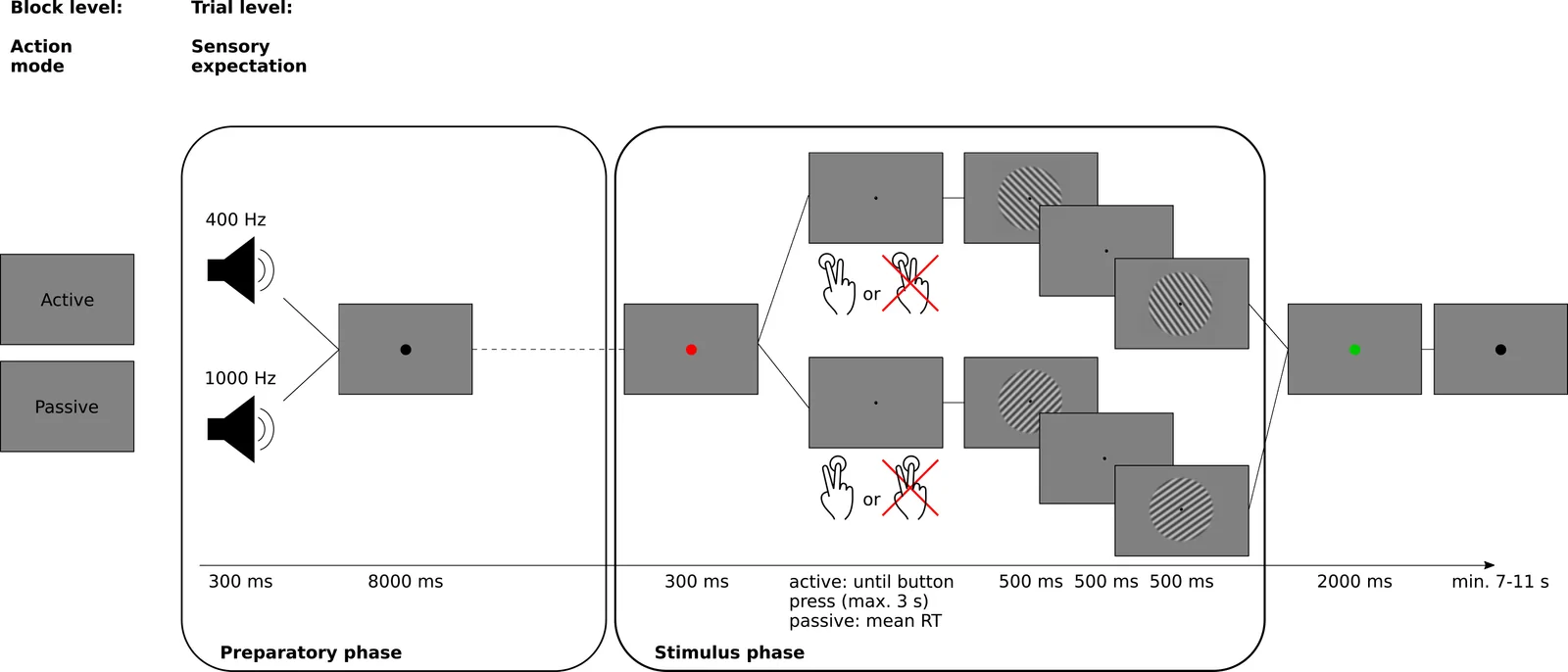
Experimental paradigm Active and passive conditions were presented in separate blocks. Each trial started with a predictive cue indicating the overall orientation of the upcoming gratings and informing participants which button to press in the active condition. After the cue, a preparatory phase started, during which only a fixation dot was shown. Then the fixation dot briefly turned red, which meant that participants had to now press a button with their index or middle finger to elicit the presentation of the gratings (active condition) or had to wait for the gratings to be presented automatically (passive condition). The waiting time in the passive condition was the average reaction time of the participant in active trials in the behavioral experiment. The two gratings differed slightly in their orientation (±0.5–6.5), and participants were asked to judge whether the second grating was tilted clockwise or counterclockwise compared with the first one. They had to report their answer when the dot turned green after grating offset. Any time that remained from the button press window in the active condition to elicit grating presentation was added to the intertrial interval (ITI) (base ITI: 7–11 s, mean 9 s). This means that trials had, on average, the same duration (24.1 s). The mapping between cue frequency, button, and grating orientation was counterbalanced across participants. In the GLM for the fMRI analyses, the preparatory phase was modeled from the onset of the auditory cue until the end of the 8,000 ms preparatory phase, just before the color change of the fixation dot. The stimulus phase was modeled from the color change of the fixation dot until the offset of the second stimulus. These phases were modeled separately for active and passive trials. The question phase was modeled in a separate regressor for both active and passive trials. The ITI was left unmodeled.

Participants performed both a behavioral and an fMRI experiment that used the same stimuli and experimental paradigm (see Figure 1). Participants had to perform an orientation judgment task on pairs of gratings that they either caused themselves to appear (active blocks) or that appeared automatically (passive blocks). Each trial started with an auditory cue that predicted with 100% validity the overall orientation of two consecutively presented gratings (45° leftward or 45° rightward). There was a preparation phase of 8 s between the auditory cue and the active/passive stimulus generation, during which only a fixation dot was presented on the screen, but participants already had a prediction about the upcoming stimuli due to the cue. We performed our fMRI analyses for this preparation phase and for the stimulus phase separately. In the behavioral experiment, the preparation phase after the cue was reduced to 3 s.

### Behavioral effects

**Figure 2:**
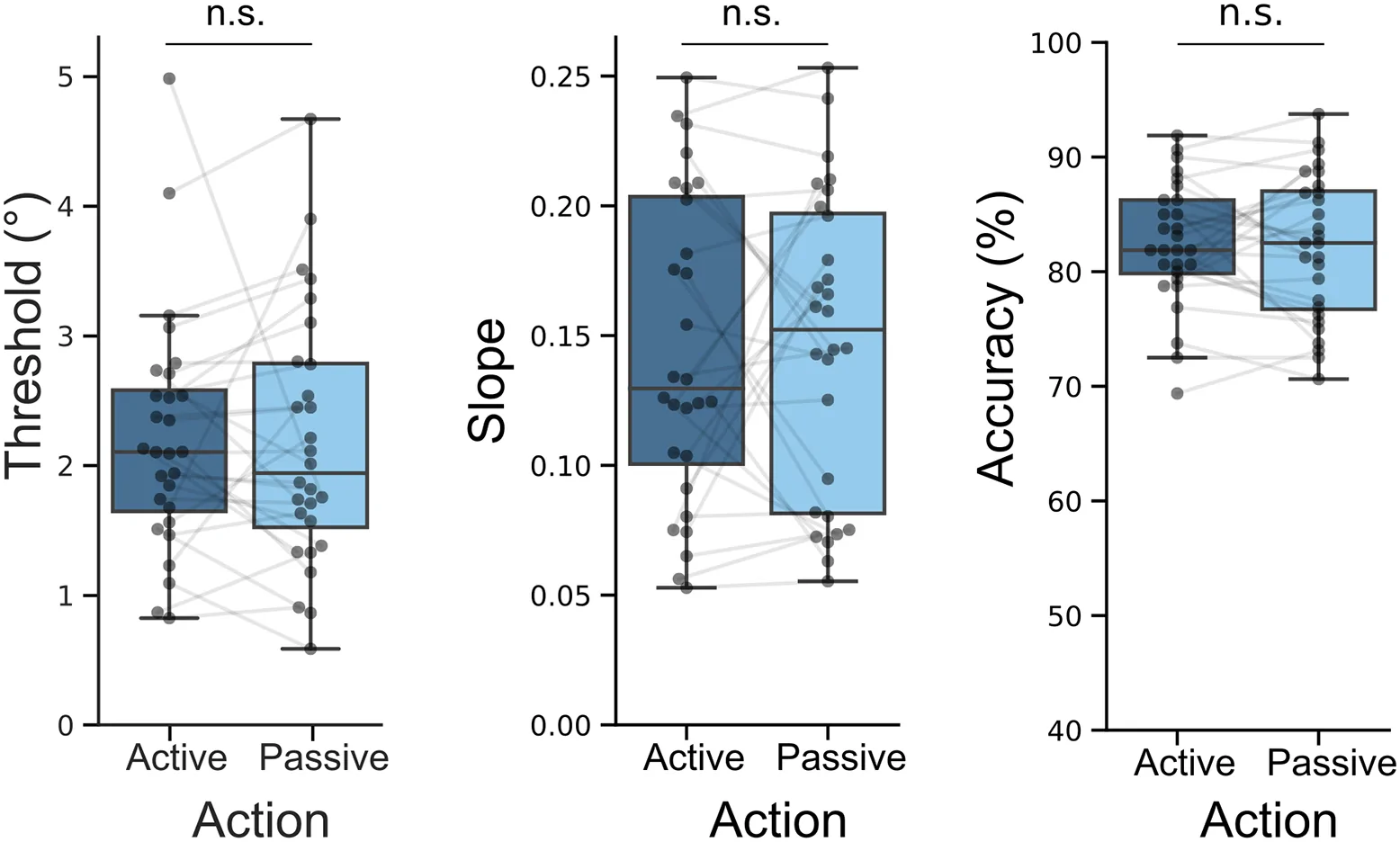
Behavioral results Boxplots showing the thresholds and slopes derived from psychometric functions for each condition, as well as the overall accuracy. n.s. = not significant.

We fitted psychometric functions to the data from the behavioral experiment for active and passive conditions separately and derived thresholds and slopes. Furthermore, we calculated the overall accuracy. Paired-samples *t* tests testing for differences between active and passive conditions revealed no significant differences, neither for thresholds (t(27) = 0.209, *p* = 0.836), nor slopes (t(27) = −0.216, *p* = 0.831), or overall accuracy (t(27) = 0.310, *p* = 0.759), as displayed in Figure 2.

**Figure 3:**
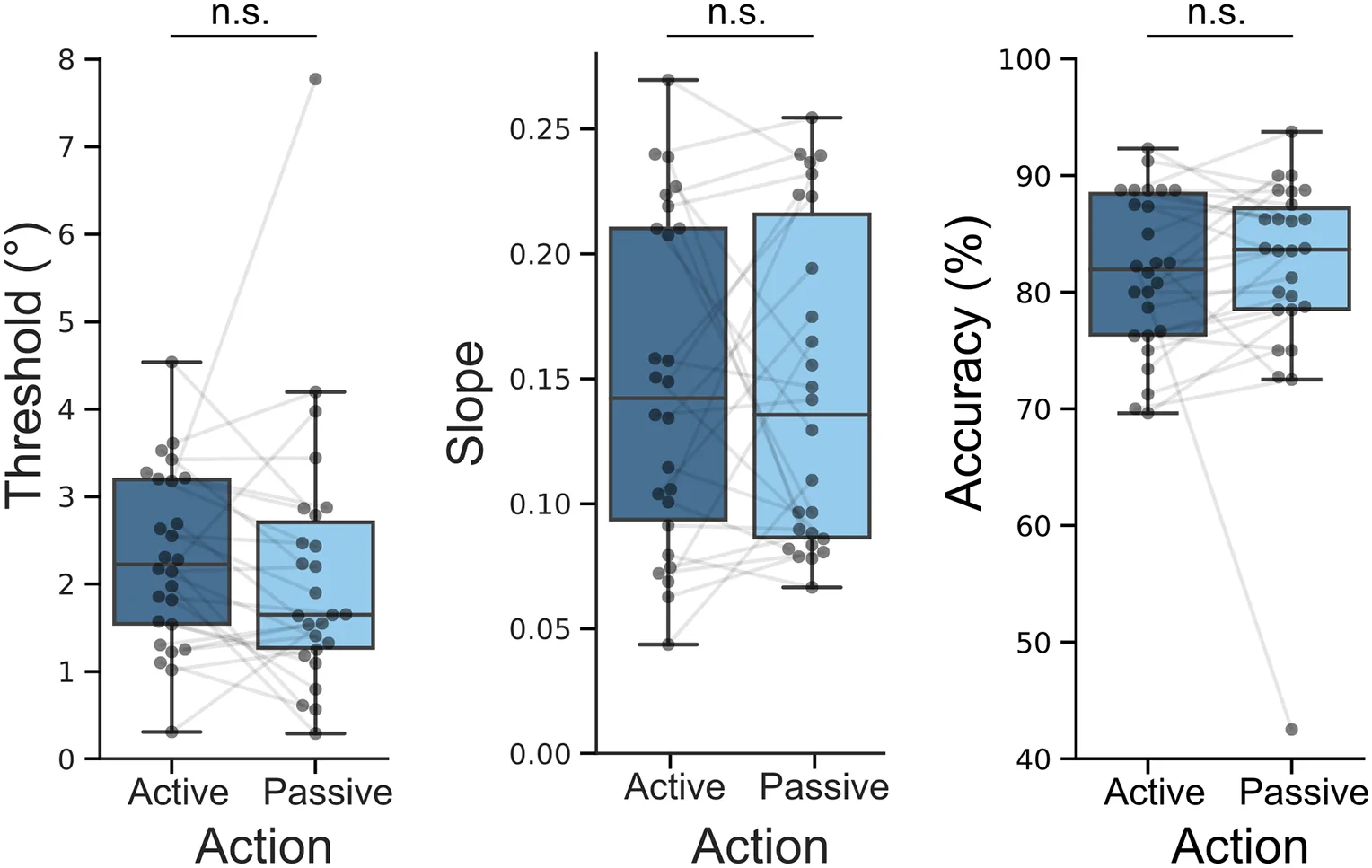
Behavioral results from the fMRI experiment Boxplots showing the thresholds and slopes derived from psychometric functions for each condition, as well as the overall accuracy. n.s. = not significant.

We applied the same approach to the behavioral data obtained during the fMRI experiment. Replicating the findings from the behavioral experiment, we found no significant differences between active and passive conditions, neither for thresholds (t(25) = 0.613, *p* = 0.546), nor slopes (t(25) = 0.155, *p* = 0.878), or overall accuracy (t(25) = 0.059, *p* = 0.954), see Figure 3.

### Univariate analyses

**Figure 4:**
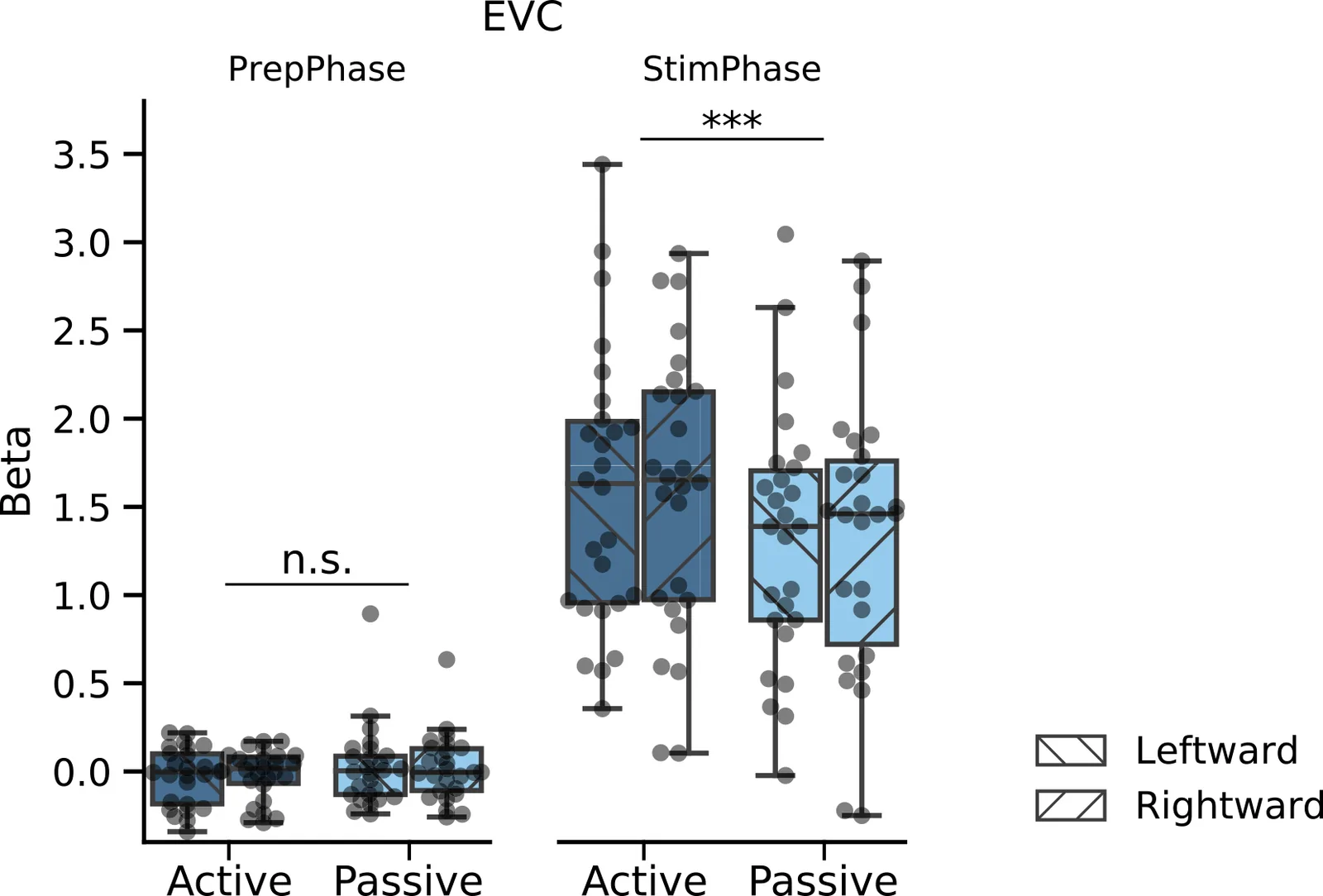
Univariate results We performed paired *t* tests to test for differences between active and passive conditions. Boxplots show the extracted beta values from early visual cortex (EVC), separate for leftward and rightward gratings. n.s., not significant; ∗∗∗*p* < 0.001.

Self-generated stimuli evoked more activity than externally generated stimuli in early visual cortex (EVC) (t(25) = −4.463, *p* = 0.00015). During preparation, no significant differences between active and passive conditions were found (t(25) = −1.392, *p* = 0.176), see Figure 4. The results were comparable across all regions of interest (ROIs) (see Figure S1). The exploratory whole-brain analysis is reported in the supplementary material (Figure S2).

**Figure 5:**
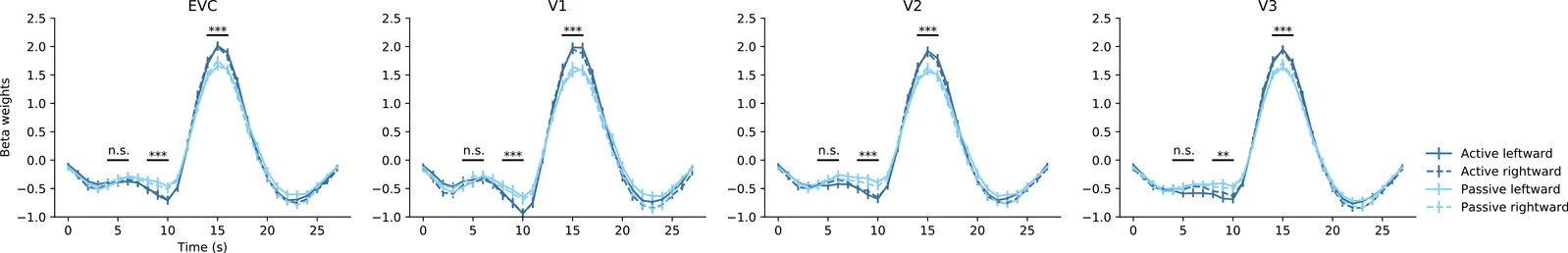
Results from deconvolution GLM Time courses are plotted for each condition (active/passive and leftward/rightward gratings), time-locked to the start of a trial (auditory cue), for early visual cortex (EVC) as well as each visual area separately. Error bars represent the standard deviation. n.s., not significant; ∗∗*p* < 0.01, ∗∗∗*p* < 0.001.

Next, in order to test whether there were any differences in the timing of the blood-oxygen-level-dependent (BOLD) response, we performed a deconvolution generalized linear model (GLM). We tested for differences between active and passive conditions in the early (time points 4–6 s) and late (time points 8–10 s) preparatory phase, as well as during the peak of the stimulus-evoked responses at time points 14–16 s. As can be seen in Figure 5, responses did not differ in their timing. Instead, amplitude differences were found in the late preparatory phase, with more sustained activity in the passive condition (V3: *p* = 0.0018; all other *p* < 0.001), and in the stimulus phase, with a larger response in the active condition (all *p* > 0.001). Responses did not differ in amplitude in the early preparatory phase (EVC: t(25) = −1.109, *p* = 0.267; V1: t(25) = −0.724, *p* = 0.469; V2: t(25) = −1.699, *p* = 0.089; V3: t(25) = −1.870, *p* = 0.062).

Our analysis of eye-tracking data showed that the differences between active and passive conditions cannot be explained by differences in fixation behavior, neither during the preparatory phase (t(13) = −1.345, *p* = 0.202) nor during the stimulus phase (t(13) = −1.752, *p* = 0.103).

### Multivariate analyses

**Figure 6:**
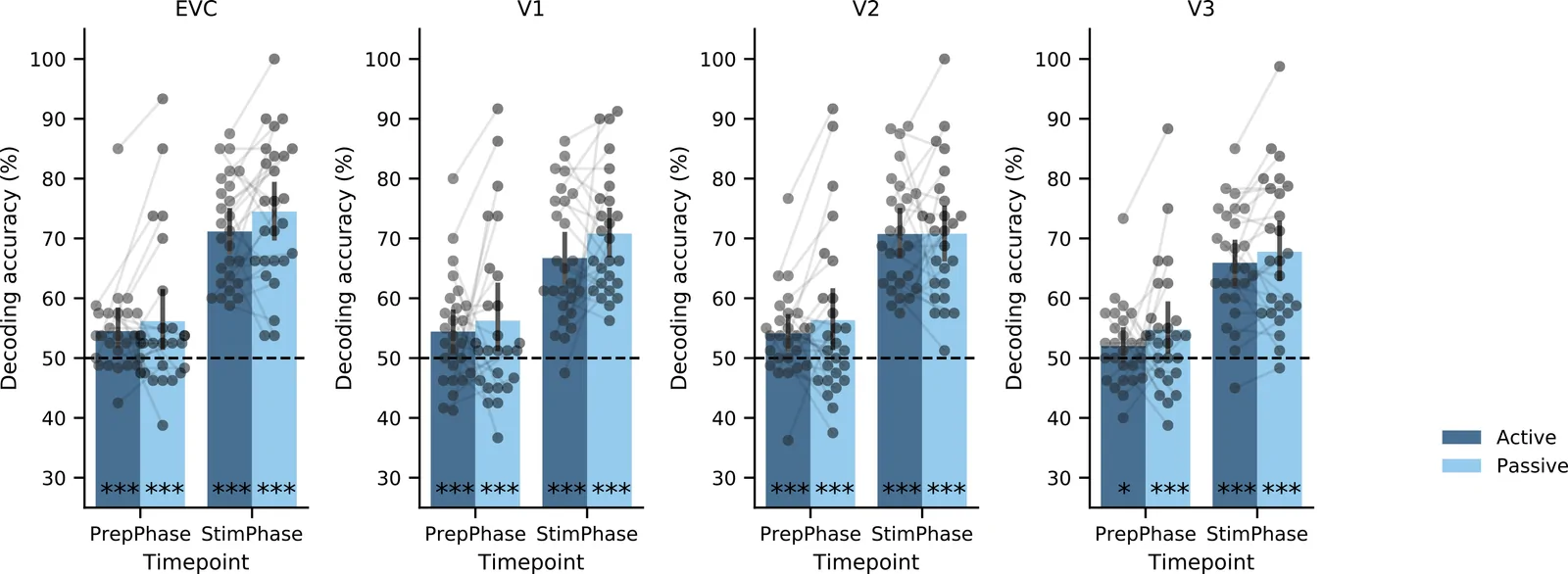
Results from multivariate analysis Decoding accuracy is plotted for each single participant. Error bars represent the 95% confidence interval. Chance level is indicated by the dotted line at 50%. ∗*p* < 0.05, ∗∗∗*p* < 0.001.

Figure 6 illustrates the results of our multivariate analyses. The orientation of the gratings could be decoded significantly above chance during the stimulus phase in all ROIs (active conditions: EVC 71.2%, V1 66.7%, V2 70.7%, V3 65.9%; passive conditions: EVC 74.5%, V1 70.8%, V2 70.8%, and V3 67.8%; all *p* < 0.001), as was to be expected based on previous studies.^18^ Importantly, the orientation of upcoming, not yet presented stimuli in the preparatory phase could also be decoded significantly above chance, again in all visual areas (active conditions: EVC 54.5%, V1 54.4%, V2 54.1%, and V3 52.0%; passive conditions: EVC 56.2%, V1 56.3%, V2 56.3%, and V3 54.8%; V3 active conditions *p* = 0.025; all other *p* < 0.001). There were no significant differences between active and passive conditions in any visual area, neither during the preparatory nor the stimulus phase (PrepPhase: all *p* > 0.195; StimPhase: EVC t(25) = −1.723, *p* = 0.097; V1 t(25) = −1.840, *p* = 0.078; V2 t(25) = −0.022, *p* = 0.983; V3 t(25) = −1.247, *p* = 0.224).

**Figure 7:**
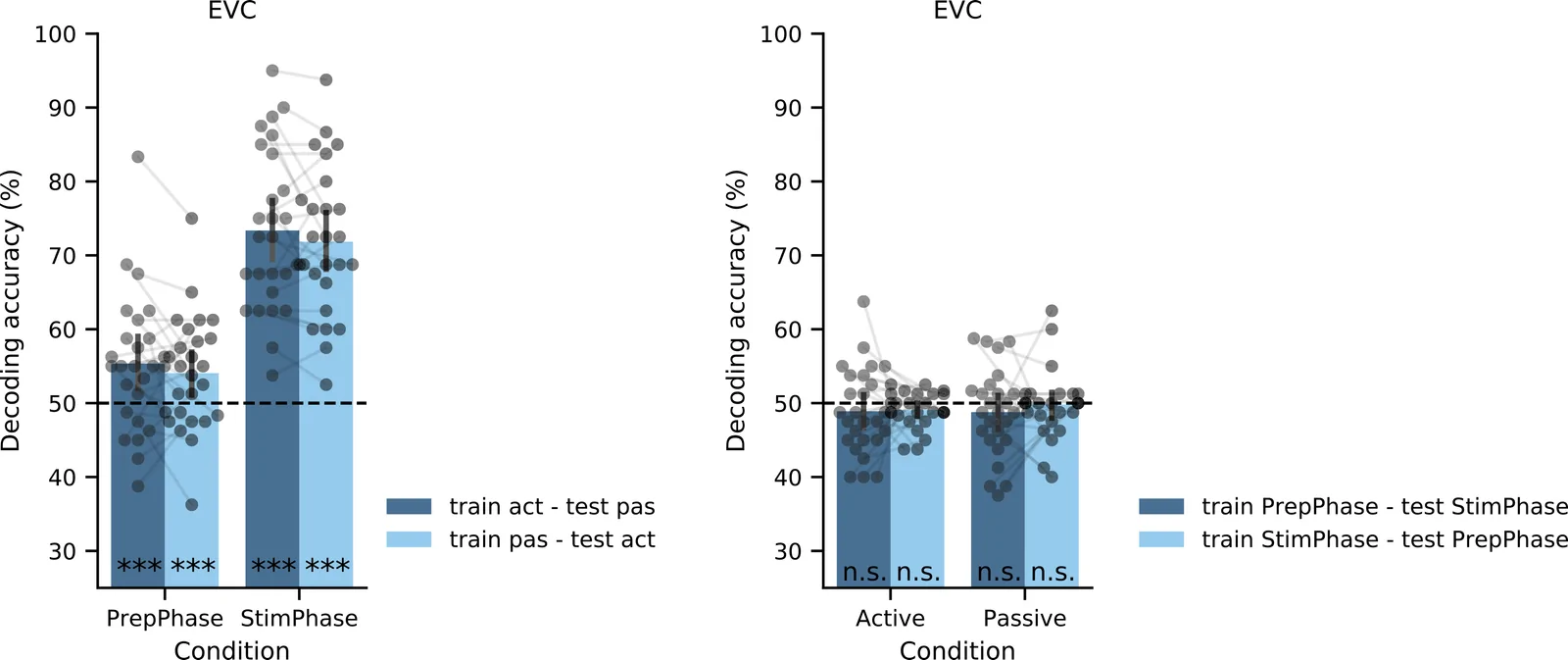
Results from multivariate analysis, cross-classification Decoding accuracy is plotted for each single participant. The image on the left shows results for training on active and testing on passive conditions (train act-test pas) and vice versa (train pas-test act). The image on the right shows results for training on the preparatory phase and testing on the stimulus phase (train PrepPhase-test StimPhase) and vice versa (train StimPhase-test PrepPhase). Error bars represent the 95% confidence interval. Chance level is indicated by the dotted line at 50%. n.s., not significant; ∗∗∗*p* < 0.001.

In order to test for similarities between active and passive conditions, we performed cross-classifications (Figure 7). Training the classifier on active trials and testing on passive trials (and vice versa) revealed very similar results, showing significant above-chance decoding in both the preparatory and stimulus phase, indicating the classifier could generalize across conditions (training on active and testing on passive trials, PrepPhase: 55.4%, StimPhase: 73.4%; training on passive and testing on active trials, PrepPhase: 54.1%, StimPhase: 71.8%; all *p* < 0.001). In contrast, training on the preparatory phase and testing on the stimulus phase (and vice versa) showed chance-level decoding for both active and passive conditions, suggesting that the underlying patterns differed between these time points (training on the PrepPhase and testing on the StimPhase, active conditions: 48.9%, *p* = 0.869, passive conditions: 48.8%, *p* = 0.892; training on the StimPhase and testing on the PrepPhase, active conditions: 49.1%, *p* = 0.941, passive conditions: 49.8%, *p* = 0.620). Results were comparable across all ROIs (see Figures S3 and S4).

## Discussion

This study investigated how action-based predictions modulate activity in EVC. Overall, our results do not support the cancellation, sharpening, and pre-activation accounts. Instead, our results mostly align with the opposing process theory (see Table 2). In line with our predictions for this theory, we show that both action-based and general sensory predictions induce similar stimulus-specific representations prior to stimulus presentation, as evidenced by significant decoding and cross-decoding. During stimulus presentation, we also found no differences in decoding accuracy between conditions, and cross-classification between conditions was possible. However, we observed enhanced, rather than suppressed, BOLD responses for self-generated stimuli, a finding inconsistent with all the aforementioned theories. Altogether, these results suggest that (1) action-based and general sensory predictions are based on similar mechanisms and that (2) action execution can boost the amplitude, but not precision, of neural responses.

**Table 2:** Results compared with the hypotheses from each theory

	Preparatory activity	Preparatory decoding	Stimulus activity	Stimulus decoding
Forward model	−	−	A+ < P+	A+ < P+
Sharpening	−	−	A+ < P+	A+ > P+
Pre-activation	A+ > P	A+ > P	A+ < P+	A+ < P+
**Opposing process**	***A+ = P+***	**A+ = P+**	A+ = P+	**A+ = P+**
Null	***A = P***	A = P	A = P	**A+ = P+**

**Results**	**A = < P**	**A+ = P+**	**A+ > P**	**A+ = P+**

Matching results are written in bold; partially matching results are written in bold italics. The above-chance decoding during the stimulus phase, with no differences between active and passive conditions, matches the predictions made by the opposing process theory and the null theory. The above-chance decoding during the preparatory phase is unique to the opposing process theory. The univariate results during the preparatory phase partly match the opposing process predictions as well as the null theory. Only the enhancement during the stimulus phase does not match any prediction.

Our finding of enhanced rather than suppressed responses to self-generated stimuli challenges the forward model, also known as the cancellation account, which posits that efference-copy-based predictions suppress sensory consequences of actions. Although this theory has strong behavioral and neural support, showing reduced perceived intensity and neural responses for self-generated stimuli,^6^^,^^7^^,^^8^^,^^10^^,^^19^^,^^20^^,^^21^^,^^22^^,^^23^^,^^24^^,^^25^^,^^26^^,^^27^ we found no behavioral differences in orientation performance and an increased BOLD response for self-generated stimuli. Our findings align with other studies reporting neural enhancement.^28^^,^^29^^,^^30^^,^^31^ It is still unclear why action sometimes suppresses and sometimes enhances neural activity. It is possible that the effect of action depends on task demands. In most studies showing suppression, participants had either no task at all or had to judge the intensity of the stimuli. In the studies showing enhanced neural responses, participants had to closely monitor their actions, as they either had to play tones in the correct order or time^30^^,^^31^ or had to reproduce a specific distance traveled.^29^ Although our study did not require close action monitoring, participants performed a demanding discrimination task. Having control over when the stimuli would appear in the active condition may have increased attention and boosted neural responses in visual cortex. Variability across sensory modalities may also be relevant: suppression is more consistent in tactile and auditory domains, whereas visual studies report mixed effects. For example, while Cardoso-leite et al.^23^ found reduced sensitivity for self-generated visual stimuli, Schwarz et al.^32^ failed to replicate this result. Other behavioral studies show either enhancement or suppression depending on task timing.^33^^,^^34^ On the neural level, studies report reduced responses in visual areas,^7^^,^^10^^,^^35^^,^^36^^,^^37^^,^^38^ enhanced responses,^39^^,^^40^^,^^41^ or mixed effects depending on participants or event-related potential (ERP) components.^28^^,^^42^ These heterogeneous results for the visual modality compared with the tactile and auditory modality point at the possibility of modality-specific differences. Alternatively, differences in the type of stimulus (body-related and proximal vs. abstract and distal) used in these studies may explain some of these differences (see Dogge et al.^43^). Another factor that may play a role is temporal predictability. In the majority of previous studies, an active condition was usually compared with a condition in which stimuli were presented automatically at jittered time points. Therefore, the exact time point at which the stimulus would be presented could not be predicted, whereas in an active condition, participants always knew when the stimulus occurs, since they can perfectly plan when to execute the action and, therefore, predict the temporal occurrence of the stimulus. The conditions would, therefore, differ in their temporal predictability, which could (partly) explain sensory suppression. This is a confound that has been criticized before.^44^ In order to control for this, we kept the stimulus onset perfectly predictable, by presenting it at a fixed interval after the cue. In doing so, we may have significantly reduced or even abolished the sensory suppression effect. In turn, differences in attentional resources may have become more apparent. In the active condition, participants had to pay close attention to know when they could press the button and check whether their button press was successful. In contrast, the passive condition was so predictable that it, therefore, may have required less attention, thus leading to less neural activity. Our study cannot determine the cause of the enhanced BOLD response in visual cortex for actively generated stimuli and the lack of behavioral differences. Future research should explore different tasks to test how task demands, sensory modalities, stimulus predictability, and attention interact to drive suppression or enhancement of self-generated stimuli. Thus, we cannot completely reject the forward model; all we can say is that with our current design, we could not find any evidence for sensory suppression as posed by the forward model theory.

It should be noted that most studies showing sensory suppression presented stimuli for 300 ms or less. This is clearly shorter than in our study, in which we presented two stimuli sequentially with 500 ms duration each, separated by an interval of 500 ms. The stimulus phase, therefore, totaled 1,500 ms. Sensory suppression is characterized by temporal precision, in the sense that even short delays of <400 ms between action and outcome can diminish or even eliminate the suppression effect. In our study, stimuli were always presented immediately after the button press without a delay. There should, therefore, not have been any temporal prediction errors that reduce or eliminate suppression. However, as our stimuli were presented for longer than in most studies, we cannot rule out that suppression may have occurred only at the start of stimulus presentation. Such short-lived effects are often nonetheless detectable with fMRI; an example in which we were able to detect an adaptation effect that only happens in one frame of a sequence of images convolved in a longer BOLD response is Weigelt et al.^45^ However, when modeling the whole stimulus phase, a short-lived suppression may not sufficiently affect the average activation elicited by the long stimulation. Our specific paradigm is based on that used by Kok et al.,^13^ who studied the influence of sensory predictions outside the context of action and also used two gratings that were presented for 500 ms each with a 500 ms interval. Despite these long durations, they also observed BOLD suppression for expected stimuli. Nevertheless, with the same paradigm, we found BOLD enhancement instead. Together, it seems unlikely that there was suppression that we failed to observe. Nonetheless, the longer stimulus duration could have obscured initial suppression effects.

Our study also found no support for the pre-activation account, which proposes that learned action effects pre-activate sensory regions, resulting in early activation followed by perceptual suppression.^11^ Several behavioral studies support this idea, showing early facilitation and later suppression of perception of self-generated stimuli.^11^^,^^33^^,^^34^ Furthermore, it has been reported that active movements lead to an earlier BOLD response than passive movements.^46^ However, our univariate GLM could not find any significant differences between active and passive conditions in the preparatory phase. A deconvolution GLM revealed divergence just before stimulus onset, but with stronger sustained activity in the passive condition. It is possible that this stronger sustained activity is related to differences in attentional demands. In the passive condition, the participant is continuously tracking the display to not miss the stimulation onset, whereas in the active condition, the participant controls the onset of the stimulus through their own button press. They, therefore, do not risk missing the stimulus presentation in the active condition and only need to check whether the fixation dot turns red to know when they can press the button, as compared with sustaining attention in the passive condition. Alternatively, the differences in activity relate to a stronger initial dip in the active condition. If a stronger initial dip at the end of the preparatory phase is taken as evidence for more activity, then there would be some indirect support to the pre-activation model. As we cannot tease apart these explanations with the current study, these results need to be interpreted with caution. Multivoxel pattern analysis showed that the orientation of predicted upcoming gratings could be decoded from preparatory visual cortex activity. This finding aligns with previous research showing that predictions outside the context of action induce stimulus representations prior to their occurrence^17^ or during omission of an expected stimulus.^16^ However, decoding performance was similar across conditions, and significant cross-classification suggests shared neural patterns. While electroencephalogram (EEG) studies have reported stronger pre-activation for active conditions a few hundred milliseconds prior to action execution,^47^^,^^48^ these short-lived effects may have been undetectable with fMRI. Overall, our results suggest that both action-based and general sensory predictions lead to similar pre-activation in early visual regions. This challenges the idea that action uniquely enhances pre-activation, highlighting the need for further investigation into the interplay between action, prediction, and neural dynamics.

The sharpening account was also not supported by our results. The concept of predictions leading to sharpening originates from the general perception literature. Kok et al.^13^ demonstrated that expected stimuli result in a reduced neural response yet improved decoding performance. This finding suggests that expectations sharpen the neural response, an effect that may appear as suppression when examined using univariate measures. Yon et al.^14^ extended this analysis to self-generated stimuli and observed similar results: expected sensory action outcomes led to reduced activity but improved decoding compared with unexpected outcomes. These findings were taken as evidence that action does not suppress neural activity, but instead sharpens neural responses. However, we found no difference in decoding accuracy between active and passive conditions. Unlike Yon et al.,^14^ who compared expected and unexpected action outcomes (all self-generated), we compared self-generated and externally generated stimuli, both of which were expected and temporally predictable. The absence of differences in decoding performance between conditions suggests that action-based and general predictions may operate similarly under predictable conditions. Since our study did not include unexpected events, we cannot confirm whether predictions lead to a sharpening of neural responses for expected compared with unexpected stimuli. However, our results indicate that action-based predictions do not sharpen neural responses more than general sensory predictions do.

Overall, our results mostly align with the opposing process theory.^15^ We observed pre-activation for both action-based and general predictions, and no difference in decoding accuracy during stimulus presentation—consistent with the theory’s prediction for expected stimuli. However, we did find enhanced BOLD responses for self-generated stimuli, which the theory does not fully explain in this context. One possibility is that nonspecific factors, like increased attention or arousal associated with action, boosted neural responses. Another explanation might be linked to motor-induced responses in the visual cortex, as observed, for example, in mice^49^^,^^50^^,^^51^ (for an overview, see Schneider^52^). Lastly, studies have found that action modulates visual perception in a rhythmic fashion, with sensitivity oscillating at theta band frequencies.^53^^,^^54^^,^^55^ If self-generated stimuli are associated with stronger phase-locking than externally triggered stimuli, as reported by Benedetto et al.,^53^ this may be an additional explanation for the BOLD enhancement we observed in visual cortex. As our study was not designed to capture the above-mentioned phenomena, we cannot rule out the influence of these additional processes.

Altogether, we have shown that action-based and general sensory predictions pre-activate neurons in visual cortex by inducing a representation of the predicted upcoming stimulus. These representations were similar for self-generated and externally generated stimuli, suggesting similar mechanisms. Furthermore, we have shown that action can boost the amplitude, but not precision, of neural responses to self-generated stimuli. These results fail to support both the forward model theory, as well as the pre-activation and sharpening accounts. The similar pre-activation is in line with the opposing process theory (Press et al., 2019),^15^ but the BOLD enhancement is not predicted by any of these theories, and may be driven by attentional factors that need further research.

### Limitations of the study

As mentioned in the discussion, there are some limitations of the study. Firstly, by keeping the passive condition temporally predictable in order to control for differences in temporal predictability between active and passive conditions, we may have reduced sensory attenuation effects. Attentional demands may have differed between the conditions due to the inherent nature of having to perform an action in the active condition, which is absent in the passive condition. Furthermore, our stimuli were presented for longer than in most studies, and due to the sluggish nature of fMRI, we can, therefore, not resolve brief suppression effects at the start of the stimulus if they are canceled out by later compensatory processes. Lastly, since we did not include unpredictable events, we can directly test the forward model, sharpening, and pre-activation but only partly test the opposing process theory. Our results, therefore, cannot clearly reject the forward model, the sharpening, and the pre-activation accounts. However, our results do point at overlapping mechanisms between action-based and general sensory predictions, at least when stimuli are equally predictable, aligning with the opposing process theory. Future studies should manipulate the predictability of stimuli as well as attention, stimulus duration, and timing with regard to action onset to further disentangle these effects.

## Resource availability

### Lead contact

Requests for further information and resources should be directed to and will be fulfilled by the lead contact, Bianca M. van Kemenade (bianca.van-kemenade@psychiat.med.uni-giessen.de).

### Materials availability

A detailed description of the materials is listed in the STAR Methods section. Any further information is available from the lead contact upon request.

### Data and code availability

• Data have been deposited at Zenodo and are publicly available as of the date of publication at https://doi.org/10.5281/zenodo.20507627.
• Code has been deposited at Zenodo and is publicly available as of the date of publication at https://doi.org/10.5281/zenodo.20507627.
• For other inquiries, please contact the lead contact.

## Acknowledgments

B.M.v.K. received support from the 10.13039/501100001659Deutsche Forschungsgemeinschaft (DFG, grant KE 2016/2–1 and SFB/TRR 135, grant no. 222641018, project A10). L.F.M. has received funding for this project from the 10.13039/501100000268Biotechnology and Biological Sciences Research Council (BBSRC) BB/V010956/1 (“Layer-specific cortical feedback dynamics”) and the 10.13039/501100000265Medical Research Council (MRC: UKRI532 “Cortical layer-specific imaging of context-dependent cognitive processing”). We thank Francis Crabbe and Veronika Penkova for their support in the fMRI data collection.

## Author contributions

Conceptualization, B.M.v.K. and L.F.M.; methodology, B.M.v.K. and L.F.M.; investigation, B.M.v.K.; writing – original draft, B.M.v.K.; writing —review and editing, B.M.v.K. and L.F.M.; funding acquisition, B.M.v.K. and L.F.M.; resources, L.F.M.; supervision, L.F.M.

## Declaration of interests

The authors declare no conflict of interest.

## STAR★Methods

### Key resources table

**Table:** 

REAGENT or RESOURCE	SOURCE	IDENTIFIER
**Deposited data**		

Behavioural and fMRI data	This paper	Zenodo: https://doi.org/10.5281/zenodo.20507627
Code for experiment	This paper	Zenodo: https://doi.org/10.5281/zenodo.20507627

**Software and algorithms**		

PsychoPy 3.2.4	www.psychopy.org	RRID:SCR_006571
BrainVoyager 22.0 for Linux	Brain Innovation	RRID:SCR_013057
Matlab R2019b	Mathworks	RRID:SCR_001622
LibSVM	http://www.csie.ntu.edu.tw/wcjlin/libsv	RRID:SCR_010243

### Experimental model and study participant details

A total of 28 healthy, right-handed participants were invited to take part in the study, which was comprised of three sessions: one behavioural experiment, one fMRI experiment, and one fMRI session with retinotopic mapping and functional localisers needed to create individual regions of interest. All 28 participants completed the behavioural experiment (8 male, 20 female, mean age: 23.6 years, *SD* = 4.7). Furthermore, 26 participants additionally took part in the fMRI experiment, and 25 completed all three sessions. For the participant that took part in the fMRI experiment, but not the localiser session, we were able to generate regions of interest based on the main experiment. The final sample for the fMRI study thus included 26 participants (7 male, 19 female, mean age: 23.5 years, *SD* = 4.5). For three participants, one run each had to be excluded; one due to excessive head motion, one due to technical error causing button presses to not be recorded, and one due to the participant pressing the button at incorrect times in the active condition.

### Method details

#### Stimuli

The stimuli were sinusoidal gratings with 1.5 cycles per degree (cpd), presented at 50% contrast. They were presented in an annulus (outer diameter: 8° of visual angle, inner diameter: 0.5°) surrounding a black presentation dot of 0.3°, on a midgrey background. Auditory cues were pure tones of either 400 Hz or 1000 Hz, presented through MRI-compatible headphones. Stimuli were presented with PsychoPy 3.2.4 (Peirce et al., 2019) on a Windows 10 PC. The monitor had a refresh rate of 60 Hz.

#### Experimental design

The behavioural and fMRI experiment used the same experimental design, with some small differences in timing. Each trial started with an auditory cue (400 Hz or 1000 Hz), presented for 300 ms, that predicted the overall orientation of the upcoming gratings (leftward or rightward). The mapping between the cue frequency and the grating orientation was counterbalanced across participants, and trained in the behavioural experiment prior to the fMRI experiment. Furthermore, the cue told the participants whether they had to press a button with their right index or middle finger. The association between the cue and the button was also trained in the behavioural experiment. After the auditory cue, there was a preparation phase, during which only the fixation dot was present on the screen. This phase lasted three seconds in the behavioural experiment and eight seconds in the fMRI experiment. When the preparatory phase had passed, the fixation dot turned red for 300 ms. In active conditions, this served as a cue for the participant to press the button with their right index or middle finger, after which the gratings were presented immediately as a result of the button press. If participants pressed the wrong button, nothing happened; the script waited for the participant to press the correct button. Participants were instructed to press the button within 3 seconds after the cue. In the behavioural experiment, the script nonetheless waited indefinitely until the participant pressed the button. In the fMRI experiment, the script would move on after 3 seconds and present the stimuli. This only happened in 2% of trials. In passive conditions, the fixation dot turning red signalled to participants that the gratings would be presented soon. The interval between fixation cue and presentation of gratings in the passive condition was fixed to make the timing of the stimuli predictable and thereby control for the effect of temporal predictability. In order to keep the timing in active and passive trials similar, we based the interval in passive trials on the average reaction time in active trials, which was calculated for each participant separately. In the behavioural experiment, we calculated the average time the participant took to press the button in active conditions in the training of the behavioural experiment and used this as the interval for passive trials in the main behavioural experiment for that participant. In order to account for potential training effects, we recalculated the average time the participant took using all trials of the main experiment, and used this new interval for the passive trials of the fMRI experiment. The average reaction time in the active condition was 495 ms (std: 163 ms) in the behavioural experiment, and 314 ms (std: 143 ms) in the fMRI experiment. The two gratings were presented for 500 ms each, separated by an interstimulus interval of 500 ms. The orientation of the first grating was always +45° or -45° from vertical, depending on which auditory cue was presented at the start of the trial, and the second grating deviated slightly from this orientation by one of five angles (± 0.5° - 6.5°). After the offset of the second grating, participants were asked to judge whether the second grating was oriented clockwise or counterclockwise with respect to the first grating. A green fixation dot indicated the interval during which participants could respond (behavioural: until they answered, fMRI: max. 2 s). They indicated their answer using MRI-compatible buttons (clockwise = left index finger, counterclockwise = left middle finger). In the behavioural experiment, the next trial started after an intertrial interval of 2.5 seconds. In the fMRI experiment, the next trial started after a jittered intertrial interval, with a minimum mean duration of 9 s (range: 7-11) to which remaining time from the button press intervals were added. Each run contained an active and a passive block, counterbalanced across runs, with half of the trials containing leftward and the other half containing rightward gratings. The behavioural experiment contained four runs of 80 trials each, leading to 320 trials in total. The fMRI experiment contained four runs of 40 trials each, leading to 160 trials in total.

#### Procedure

Participants were first invited to the behavioural experiment. Here, they first trained the mapping between auditory cue, button, and grating orientation, and were then familiarised with the task. They then performed a training session with the main experimental paradigm, in which only the largest angle difference (6.5°) was used, and feedback on their performance was given. If performance was below 75%, this training session was repeated. After training, all participants reached at least 75% accuracy and were able to proceed with the main experiment. The fMRI experiment took part on another day. At the start of this session, participants performed a brief training session outside the scanner to refresh their memory. They then proceeded with the fMRI experiment inside the scanner. On a third day, retinotopic mapping and functional localiser data were collected.

#### MRI acquisition

Functional MRI data were acquired using a 3T TIM Trio scanner (Siemens, Erlangen, Germany), using a 32-channel head coil. A multiband sequence was used to collect whole-brain functional data with the following settings: 45 slices interleaved; field of view [FoV] = 222 mm; repetition time [TR] = 1000 ms; echo time [TE] = 36 ms; flip angle = 60°; PAT mode = GRAPPA, multiband acceleration factor = 3, slice thickness = 3.0 mm, distance factor = 10%, and voxel size = 3 x 3 x 3 mm. A total of 995 volumes were collected per run in the main experiment, 800 volumes for the polar angle retinotopic mapping, and 196 volumes for the functional localiser. A T1-weighted anatomical scan (MPRAGE) was collected at the end of each scanning session.

### Quantification and statistical analysis

#### Behavioural analysis

We fitted psychometric functions to the behavioural data from the behavioural and fMRI experiment for active and passive conditions separately using psignifit and derived thresholds and slopes (Behavioural experiment: Figure 2; fMRI experiment: Figure 3). Furthermore, we calculated the overall accuracy. Differences between conditions were tested with paired sample t-tests.

#### MRI analysis

fMRI data were analysed using BrainVoyager 22.0 for Linux and custom Matlab scripts in Matlab R2019b. Data preprocessing included slice time correction, motion correction, high-pass temporal filtering, and coregistration to each participant‘s anatomical image. Both the anatomical and functional data were then normalised to MNI space. For the univariate analysis, functional data were additionally smoothed with 8mm. For the multivariate analysis, the unsmoothed data were used. A GLM was created with predictors modelling the two different time points of interest for each condition (active/passive and leftward/rightward gratings). The preparatory phase was modeled from the onset of the auditory cue to the end of the preparatory phase, and the stimulus phase from the onset of the first stimulus until the offset of the second stimulus. An additional predictor was included to model the time during which participants could answer in response to the orientation discrimination task, from the onset until the offset of the green fixation dot. All regressors were convolved with the canonical hemodynamic response function (HRF). For the univariate analysis, each predictor contained all trials of that condition, leading to a total of 9 predictors per run. For the multivariate analysis, each trial was modelled with a separate predictor, leading to a total of 81 predictors per run.

#### VOI definition

Visual areas V1, V2, and V3 were defined using standard retinotopic mapping procedures based on the polar angle sequence. An additional region called “early visual cortex” (EVC) was created by combining V1/2/3. Within these regions, voxels responsive to our stimuli were selected using our functional localiser (t-contrast stimulus > fixation, *p* < 0.01 uncorrected).

#### Univariate analysis

A VOI GLM was run on our regions of interest using the predictors described in *MRI analysis*. Enhancement for active trials in the preparatory phase was tested with the contrast (Active prep > Passive prep), Figure 4. We tested for suppression for active trials in the stimulus phase with the contrast (Passive stim > Active stim), Figure 4. An exploratory whole-brain univariate analysis was performed in addition using the same contrasts (see Supplementary Material). In order to test whether there were any differences in the timing of the BOLD response in our ROIs, we additionally ran a deconvolution GLM, which can be used to reconstruct the hemodynamic response function. The deconvolution analysis used a set of stick functions, one per TR, across a window of 24 seconds time-locked to the start of the auditory cue. A set of stick functions was included for active and passive trials separately. We tested differences between active and passive time courses during the early (4-6 s) and late (8-10 s) phases of the preparatory phase as well as the peak of the stimulus phase (14-16 s) with paired-samples t-tests, Figure 5.

#### MVPA

MVPA was performed with custom Matlab scripts, implementing the LibSVM software (http://www.csie.ntu.edu.tw/wcjlin/libsv). A linear support vector machine was trained to discriminate leftward from rightward gratings using the beta images resulting from the GLM. Classifier performance was tested using a leave-one-run-out cross-validation approach, and permutation testing was performed to determine statistical significance. MVPA was performed separately for active and passive conditions, as well as preparatory and stimulus phases. Paired sample t-tests were performed to determine differences in decoding accuracy between active and passive conditions, separately for the preparatory and stimulus phases, Figure 6. In addition, cross-classification analyses were performed. First, the classifier was trained on active trials and tested on passive trials, and vice versa. Second, the classifier was training on the preparatory phase and tested on the stimulus phase, and vice versa, Figure 7.

#### Eye tracking analysis

Eye movements were recorded with an MR-compatible EyeLink 1000 eyetracker system (SR Research, Osgoode, ON, Canada). We analysed the gaze coordinates during the preparatory and stimulus phase for active and passive conditions separately. The data were first smoothed with a smoothing window of 50 ms after which they were detrended and mean-corrected to remove linear drifts and correct for calibration inaccuracies. We then defined a fixation region-of-interest by a circle around the center of the screen with a radius of 1.5° and calculated the percentage of samples in which participants looked outside of this fixation area. We used paired-sample t-tests to determine whether the number of samples outside of the fixation area differed between active and passive conditions.
