# Supplementary material for: The effects of action-based predictions in early visual cortex
Source: iScience. 2026 Aug 18;29(9):117074. doi: 10.1016/j.isci.2026.117074 (PMC13520625; doi:10.1016/j.isci.2026.117074)
Supplement: Supplementary file 1 [file mmc1.pdf]

iScience, Volume *29*

Supplemental information

The effects of action-based predictions in early visual cortex

Bianca M. van Kemenade and Lars F. Muckli

Supplementary material

Results

Univariate analyses - ROI

Univariate results for each individual visual region depicted in Fig. S1 resembled the findings found in our early visual cortex ROI reported in the main manuscript. There were no significant differences between active and passive conditions during the preparatory phase in any of the visual areas (V1: t(25) = -1.634, p = 0.115; V2: t(25) = -1.425, p = 0.167; V3: t(25) = -1.034, p = 0.311). During the stimulus phase, there was significantly more activity in the active compared to the passive condition in all visual areas (V1: t(25) = -4.445, p = 0.000157; V2: t(25) = -4.439, p = 0.000159; V3: t(25) = -4.355, p = 0.000198).

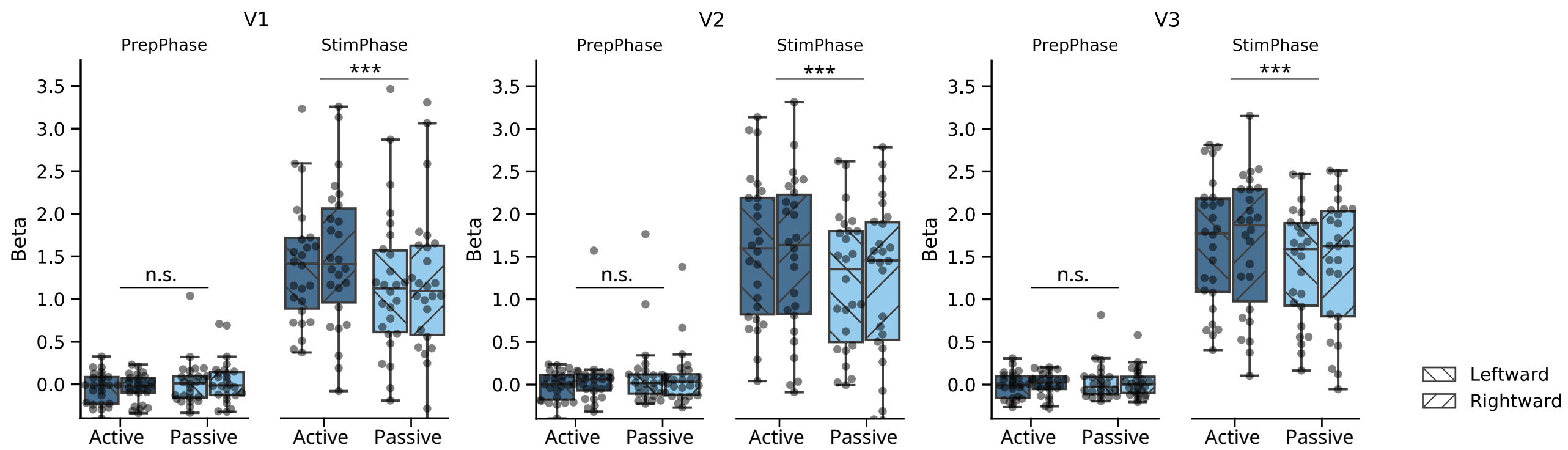

Fig. S1. Univariate ROI analysis results. *We performed paired t-tests to test for differences between active and passive conditions. Boxplots show the extracted beta values from V1, V2, and V3. n.s. = not significant, *** = p < 0.001.*

Univariate analyses – Whole-brain

Whole-brain univariate analysis showed significantly more activity for active compared to passive conditions during the preparatory phase in supplementary motor area, left primary motor cortex, and right cerebellum (Fig. S2A). These areas are typically involved in motor preparation and therefore expected, as participants were preparing to press a button with their right hand in active conditions during this phase. Aligning with our ROI analysis, no significant differences were found in visual cortex. During the stimulus phase, we observed significantly more activity for active conditions in a large network with peaks in the cerebellum, supplementary motor area, primary motor and somatosensory cortex, middle and inferior frontal gyrus, insula, and precuneus (Fig. S2B). As participants pressed a button in active conditions during this phase, it is no surprise that many areas related to motor execution were observed. Supporting our ROI analysis, active conditions also enhanced activity in visual areas.

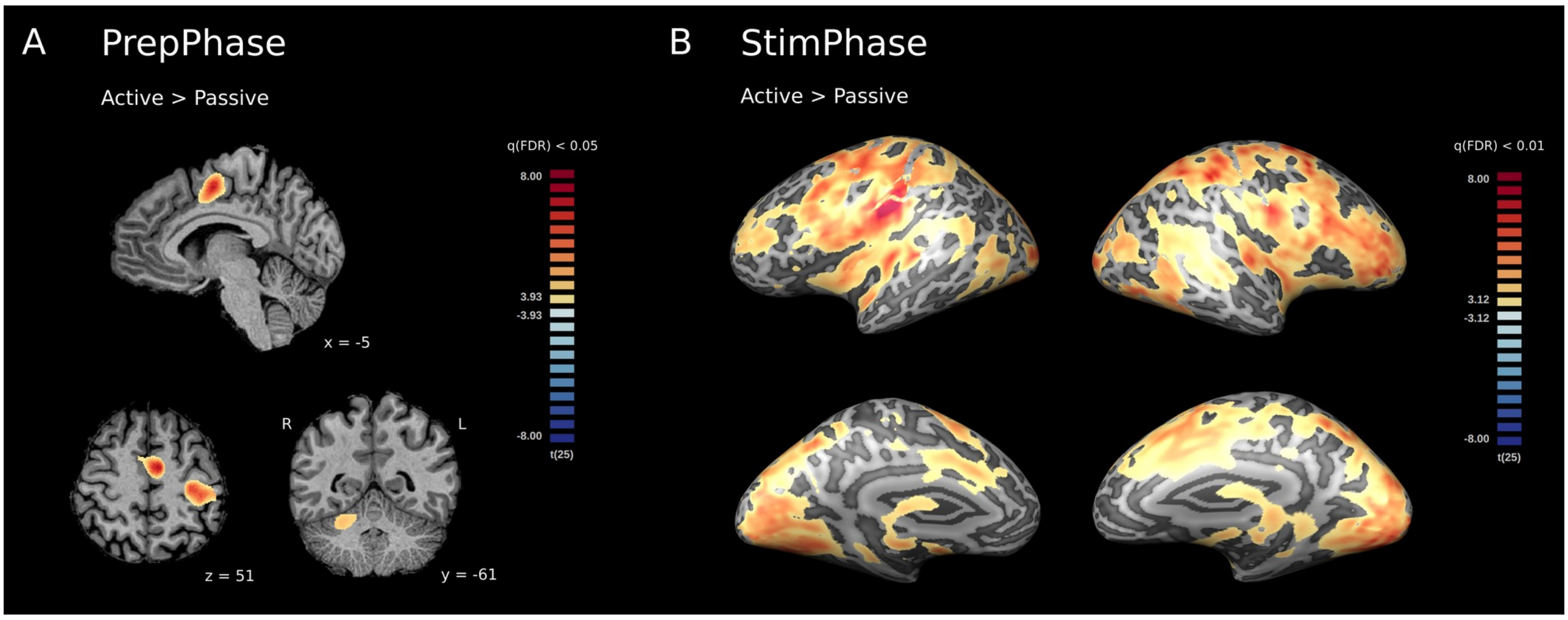

Fig. S2. Univariate whole-brain analysis results. *Active conditions were contrasted with passive conditions for the preparatory phase (PrepPhase, panel A) and for the stimulus phase (StimPhase, panel B). FDR: false discovery rate.*

Multivariate analyses – Cross-classification

Cross-classification results for each individual visual region (Fig. S3) resembled the findings found in our early visual cortex ROI reported in the main manuscript. Training the classifier on active trials and testing on passive trials (and vice versa) showed significant above-chance decoding in both the preparatory and stimulus phase, indicating the classifier could generalise across conditions (Training on active and testing on passive trials, PrepPhase: V1 52.5%, $p = 0.011$, V2 54.2%, $p = 0.002$, V3 53.0%, $p = 0.006$; StimPhase: V1 67.5%, $p < 0.001$, V2 69.7%, $p < 0.001$, V3 67.9%, $p < 0.001$. Training on passive and testing on active trials, PrepPhase: V1 54.4%, $p < 0.001$, V2 54.2%, $p< 0.001$, V3 53.0%, $p = 0.005$; StimPhase: V1 66.6%, $p < 0.001$, V2 69.7%, $p < 0.001$, V3 64.8%, $p < 0.001$).

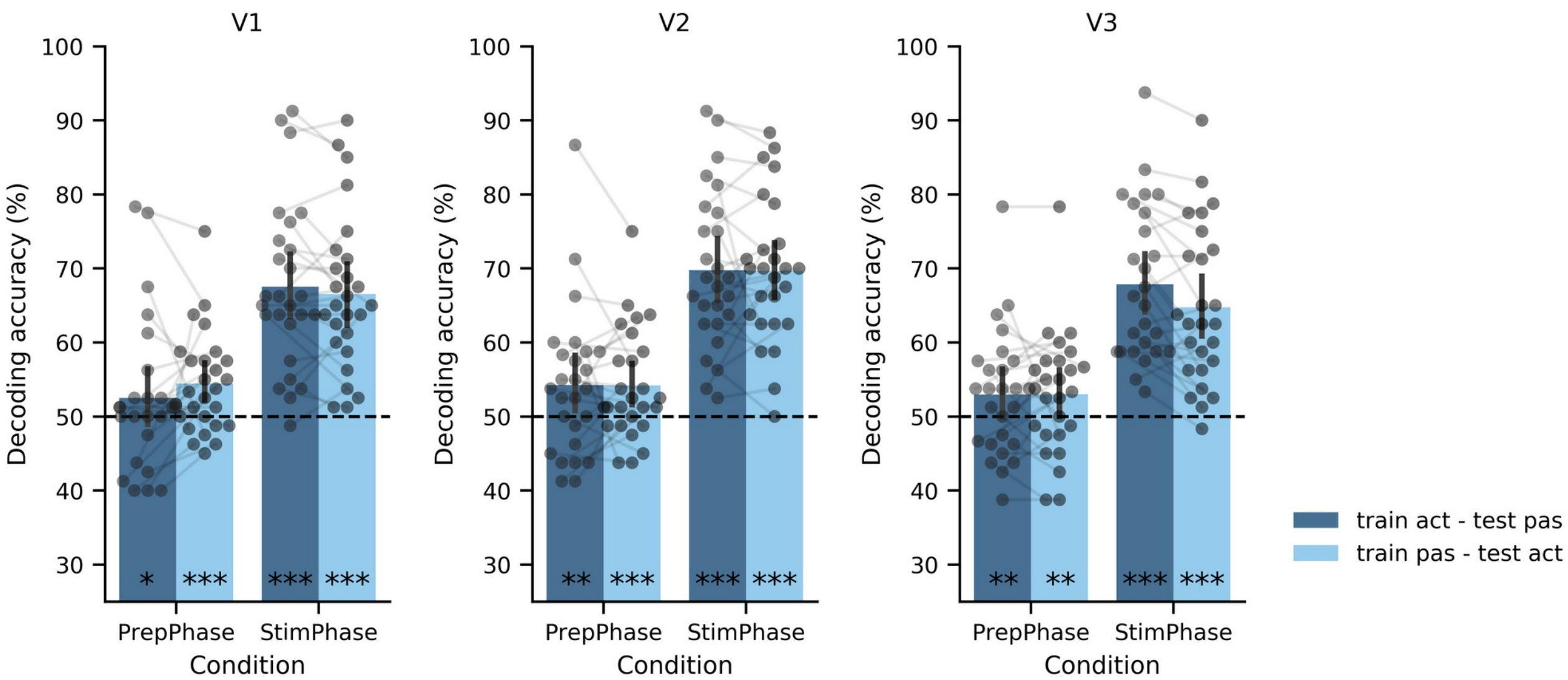

Fig. S3. Results from multivariate analysis, cross-classification, for areas V1, V2, and V3. *These are the results for training on active and testing on passive conditions (train act – test pas) and vice versa (train pas – test act). Decoding accuracy is plotted for each single participant. Error bars represent the 95% confidence interval. Chance level is indicated by the dotted line at 50%. * = p < 0.05, ** = p < 0.01, *** = p < 0.001.*

In contrast, training on the preparatory phase and testing on the stimulus phase (and vice versa) showed chance-level decoding for both active and passive conditions (Fig. S4), suggesting that the underlying patterns differed between these time points (Training on PrepPhase and testing on StimPhase, active conditions: V1 47.5%, p = 0.998, V2 48.7%, p = 0.902, V3 48.7%, p = 0.915; passive conditions: V1 48.6%, p = 0.910, V2 49.8%, p = 0.584, V3 48.4%, p = 0.938. Training on StimPhase and testing on PrepPhase, active conditions: V1 49.2%, p = 0.904, V2 49.6%, p = 0.778, V3 49.3%, p = 0.839; passive conditions: V1 51.1%, p = 0.03, V2 49.5%, p = 0.759, V3 49.3%, p = 0.871).

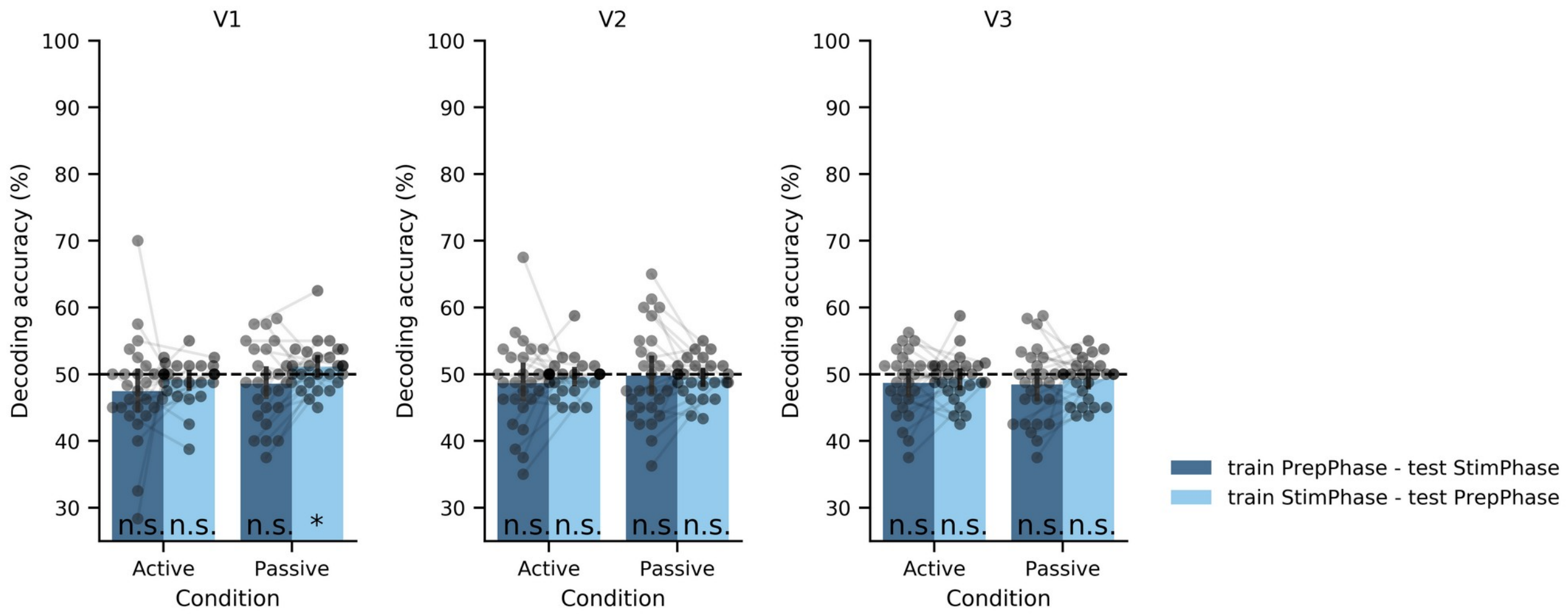

Fig. S4. Results from multivariate analysis, cross-classification, for areas V1, V2, and V3. *These are the results for training on the preparatory phase and testing on the stimulus phase (train PrepPhase – test StimPhase) and vice versa (train StimPhase – test PrepPhase). Decoding accuracy is plotted for each single participant. Error bars represent the 95% confidence interval. Chance level is indicated by the dotted line at 50%. n.s. = not significant, * = p < 0.05.*
